# Metastatic Cerebellar Gastrointestinal Stromal Tumor with Obstructive Hydrocephalus Arising from the Small Intestine: A Case Report and Review of the Literature

**DOI:** 10.1155/2014/343178

**Published:** 2014-12-28

**Authors:** Kunitomo Sato, Toshihide Tanaka, Naoki Kato, Takuya Ishii, Toru Terao, Yuichi Murayama

**Affiliations:** ^1^Department of Neurosurgery, Atsugi City Hospital, 1-16-36 Mizuhiki, Atsugi city, Kanagawa 243-0004, Japan; ^2^Department of Neurosurgery, Jikei University School of Medicine, Kashiwa Hospital, 163-1 Kashiwashita, Kashiwa, Chiba 277-8567, Japan; ^3^Department of Neurosurgery, Jikei University School of Medicine, 3-25-8 Nishi-Shinbashi, Minato-ku, Tokyo 105-8461, Japan

## Abstract

Gastrointestinal stromal tumor (GIST) is defined as a c-kit-positive gastrointestinal, mesenteric, or omental mesenchymal tumor that very rarely metastasizes to the brain. Metastasis to the cerebellum is particularly rare. An 80-year-old man presented with nausea and vomiting with disturbance of consciousness. Magnetic resonance imaging (MRI) revealed tumor in the cerebellar vermis causing obstructive hydrocephalus. The patient subsequently underwent midline suboccipital craniotomy, and the tumor was totally removed. Immunohistochemical analysis showed tumor cells positive for c-kit and CD34, and cerebellar metastasis of GIST was diagnosed. Postoperative radiotherapy was administered. Following surgery and radiotherapy, the patient developed ileus caused by tumor in the small intestine and underwent laparotomy for tumor removal. Following abdominal surgery, left hemiparesis and consciousness disturbance were noted. Computed tomography showed recurrent large tumor with perifocal edema in the right frontal lobe of the brain. The patient died 3 months after initial craniotomy. Intracranial metastasis of GIST is extremely rare. In cases such as the present, where the condition of the patient rapidly deteriorates and features such as rising intracranial pressure and ileus prevent the use of oral agents, molecular-targeted agents administered by intravenous infusion should be utilized.

## 1. Introduction

Gastrointestinal stromal tumor (GIST) is a mesenchymal tumor that arises from precursors of the* interstitial tissue* cells of Cajal in the gastrointestinal tract expressing c-kit and CD34 [1–3].

GIST usually metastasizes to the lungs and liver as a result of hematogenous spread and peritoneal seeding, and metastasis of GIST to the central nervous system (CNS) is extremely rare, with a dismal life prognosis. Most intracranial metastases from GIST have been located in cortical and subcortical areas with relatively easy surgical access and have usually been treated surgically followed by adjuvant therapy such as radiotherapy or chemotherapy.

The development of molecular-targeted anticancer agents is giving new hope to these patients. Imatinib mesylate is the first effective systemic therapy for locally unresectable or metastatic GIST [4]. Since being approved for use in treatment, imatinib mesylate has dramatically improved outcomes for patients with advanced GIST [5].

However, imatinib mesylate is believed to be ineffective against brain metastases, because it cannot pass through the blood-brain barrier (BBB) and does not achieve adequate levels in the brain when administered in lower concentrations [6]. As a second-line treatment, sunitinib, a multitargeted small-molecule tyrosine kinase inhibitor, has been shown to be effective and is currently approved worldwide for metastatic GIST [7, 8].

The effectiveness of radiotherapy for GISTs has not been confirmed. GISTs are considered “radioresistant” tumors due to their histological relationship to soft tissue sarcomas, which show relatively poor responsiveness to radiotherapy. Some case reports have indicated that radiotherapy can reduce the tumor burden and produce durable local control for locally advanced and metastatic tumors [9–11]. Radiotherapy can provide both objective responses and symptomatic relief without detracting dramatically from quality of life and should be considered in the multidisciplinary care of patients with locally advanced or metastatic GIST.

Here, we report the case of a patient with cerebellar metastatic GIST that was treated by surgical resection and radiation. In addition, we summarize and review the relevant literature regarding intracranial metastasis of GIST.

## 2. Case Report

**Figure 1:**
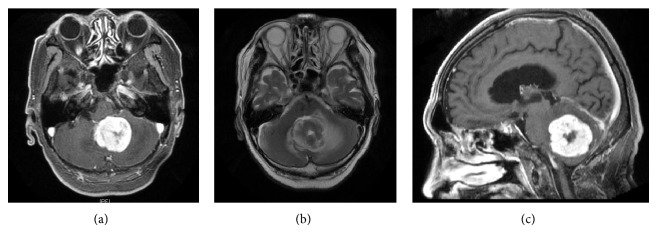
T1-weighted (a, b) and T2-weighted (c) magnetic resonance imaging (MRI) revealing the tumor in the vermis with homogeneous enhancement by gadolinium. Note that the tumor compressing medulla resulting in obstructive hydrocephalus (b).

**Figure 2:**
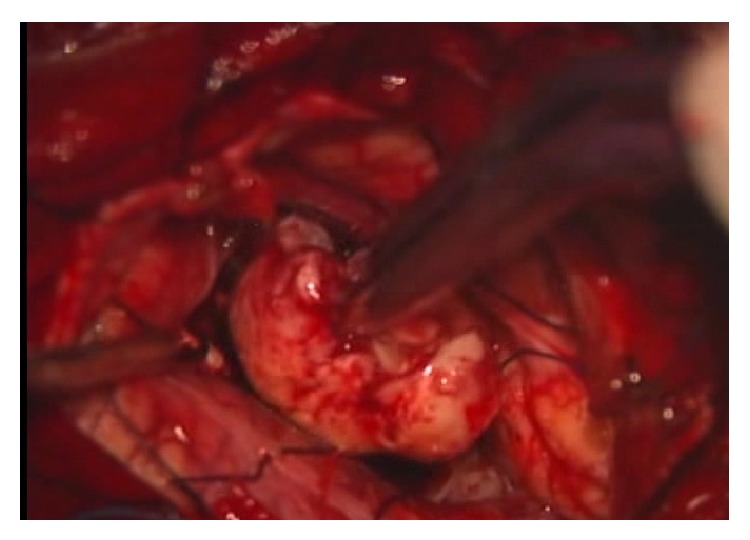
Intraoperative findings. The tumor in the vermis was totally removed. The mass appears yellowish, elastic, and hard with a clear boundary from normal structures.

The patient was an 80-year-old man who presented with nausea, anorexia, and gait disturbance. Neurological examination revealed dysmetria and dysdiadochokinesia on the left extremities. Magnetic resonance imaging (MRI) showed a round, 4-cm tumor appearing isointense on T1- and T2-weighted imaging with homogeneous enhancement (Figures 1(a)–1(c)) causing obstructive hydrocephalus. Since his condition was progressively deteriorating with consciousness disturbance rapidly, midline suboccipital craniotomy was subsequently performed to remove the tumor. The tumor was elastic, hard, and yellowish and displayed clear separation from the pia of the medulla oblongata. The tumor was totally removed (Figure 2).

**Figure 3:**
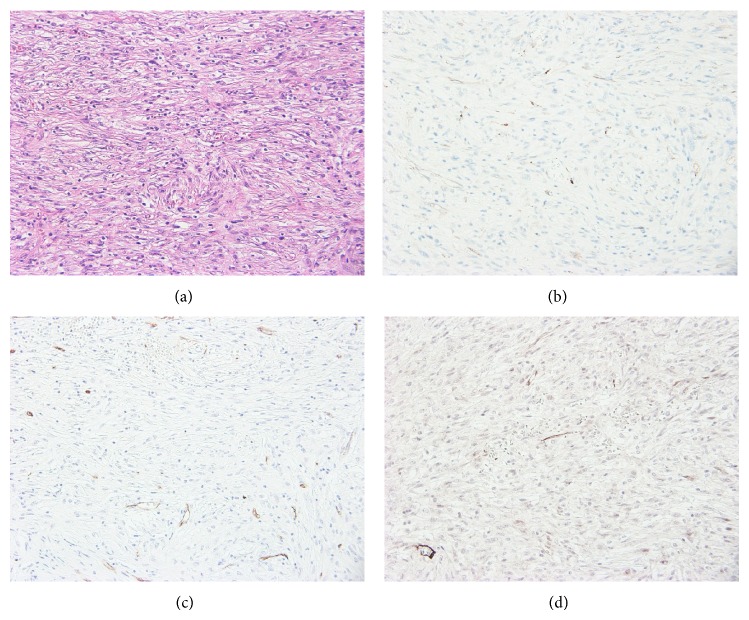
Microscopic photograph showing spindle-shaped cells with moderate cellularity crowded into bundles (hematoxylin and eosin stain, ×20) (a). Immunohistochemical studies reveal that tumor cells are positive for c-kit (b), CD34 (c), and α-smooth muscle actin (d) (×20).

Histological examination showed spindle-shaped cells with round nuclei and many mitotic figures. Spindle-shaped cells with moderate cellularity were crowded into bunches with mitotic figures (Figure 3(a)). Based on immunohistochemical studies revealing positivity for c-kit, CD34, and *α*-smooth muscle actin (Figures 3(b)–3(d)), the patient was diagnosed with GIST. At that time, no tumors were detected on endoscopic investigation of the gastrointestinal tract.

**Figure 4:**
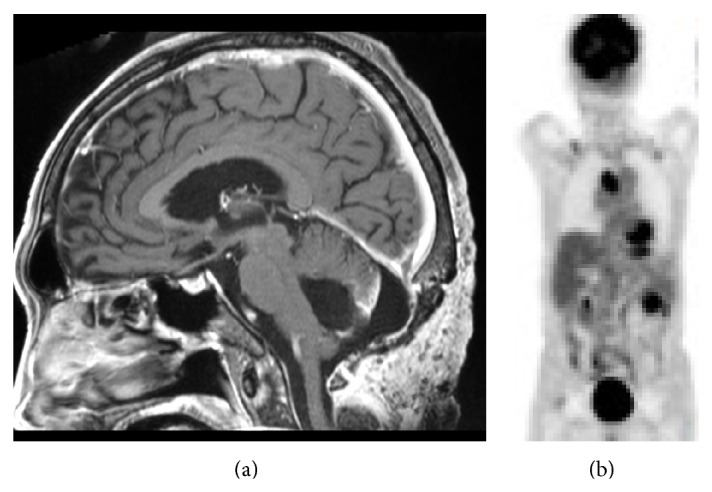
Postoperative MRI shows medulla decompressed without the residual tumor in the vermis (a). Positron emission tomography (PET) reveals abnormal accumulations in the cardiac apex, subclavial region, and peritoneal cavity (b).

**Figure 5:**
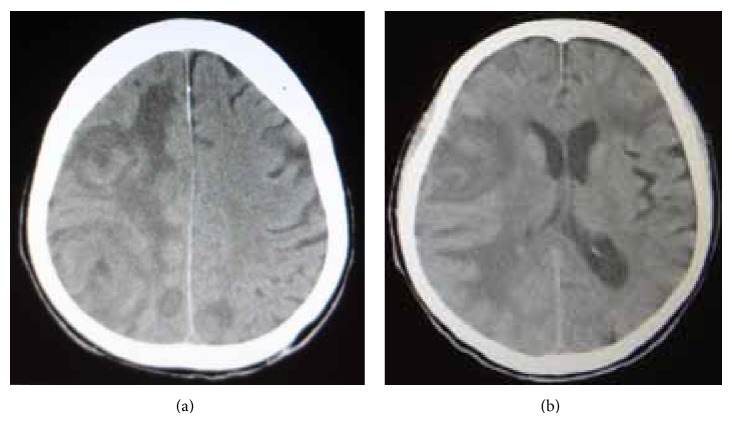
Following surgical operation and radiation therapy, computed tomography (CT) showed a large recurrent tumor with perifocal edema in the right frontal lobe (a) (b).

Postoperatively, the patient showed an improved level of consciousness and postoperative MRI showed no residual tumors (Figure 4(a)). Following craniotomy, endoscopic examination of the gastrointestine and colon showed no abnormal findings except atrophic gastritis. One month after craniotomy, radiotherapy was commenced as postoperative adjuvant therapy. Unfortunately, the patient suffered from severe abdominal pain and vomiting, which turned out to be ileus during radiotherapy. Positron emission tomography (PET) revealed multiple accumulations in the cardiac apex, subclavial region, and peritoneal cavity (Figure 4(b)). Radiotherapy was suspended (final total, 22 Gy in 11 fractions), and laparotomy was subsequently undertaken to achieve relief from the ileus with removal of the small intestinal tumor, which was identified as the primary GIST lesion. Systemic treatment with imatinib mesylate was not administered because of the patient's systemic condition. Three weeks after removal of the small intestinal tumor, the patient suffered from disturbance of consciousness with left hemiparesis. CT showed multiple recurrent tumors in the right frontal lobe with expanding perifocal edema (Figure 5). The condition of the patient gradually deteriorated and he died 4 months after initial craniotomy. Agreement for autopsy was not obtained.

## 3. Discussion

GIST mostly metastasizes within the abdomen, and the most common sites for the development of metastasis are the liver (46%) and peritoneum (41%), while concurrent GIST and metastases to the central nervous system are extremely rare.

As shown in Table 1, only 16 cases (including the present) have been reported in the literature [8–22].

The majority of metastatic intracranial GISTs are present in older adults with a median age at presentation of approximately 60 years (mean, 55.7 years). Patients have sometimes been known to show metastatic disease elsewhere prior to the development of CNS metastases. The interval between treatment of the primary lesion and diagnosis of CNS metastasis is considerably long (14 months to 12 years; mean, 4.7 years), and the prognosis is dismal once intracranial metastasis is diagnosed. Mean overall survival has been documented as 8.6 months (range, 2–35 months). In the present case, cerebellar tumor was diagnosed based on immunohistochemical findings prior to diagnosis of the primary lesion. The primary lesion turned out to be located in the small intestine and was identified when the patient suffered from ileus following craniotomy. In the three reported cases in which intracranial metastases were discovered first (including the present case), the prognosis was worse than that in those with intracranial metastases diagnosed after treatment of the primary lesion.

The current clinical practice guidelines recommend surgical resection for limited disease, particularly cortical or subcortical lesions, and adjuvant therapy as an option for patients with substantial risk of relapse [23].

Since tumor size (>5 cm) is one of the prognostic factors for aggressive behavior of GIST [24], the aim of surgery is to reduce the tumor volume to provide a cure and confirm the histological diagnosis if the metastatic tumor was discovered first, as in the present case. In such cases, surgical removal of the tumor is currently considered to be the first choice for the treatment of GIST, whether or not any distant metastases are present. Nine cases of intracranial metastasis were treated surgically. In eleven cases, patients underwent chemotherapy, including imatinib in nine patients and sunitinib in two patients. Nine patients were treated with radiation as an additional treatment. Only two of seven cases showed a response to chemotherapy. Seven patients achieved complete remission, and five of those seven patients had undergone surgery.

Recently, however, imatinib mesylate has been introduced as a molecular-targeted chemotherapeutic agent. Imatinib mesylate is an orally administered selective inhibitor of certain tyrosine kinases, including c-kit. Activating mutation of KIT or PDGFRA is found in the vast majority of GISTs, and the mutational status of these oncoproteins can be predictive of the clinical response to imatinib therapy [2, 5, 25, 26]. The prognosis of GIST treated with imatinib mesylate has recently been reported. Fletcher et al. reported age, MIB-1 index, and c-kit genetic mutation type as determining prognostic factors, whereas mutations in exon 11 of the c-kit gene correlate with the clinical outcomes of patients affected with GIST [24]. Although the genetic status of the primary lesion and CNS metastases is of interest, whether identical mutations were present in the primary and intracranial metastatic lesions remains unknown. Only five reports have described molecular features of metastatic intracranial GIST [10, 11, 16, 18, 19], with only two patients showing KIT mutation (exon 9 and exon 11) [10, 16]. Molecular findings of the primary and intracranial metastases were identical in both cases. The ability to identify the responsible gene in GISTs when KIT and PDGFRA are not mutated may lead to novel insights into the nature of GIST and new approaches to diagnosis and therapy.

Following resistance to imatinib mesylate, sunitinib, a multitargeted small-molecule tyrosine kinase inhibitor that selectively blocks vascular endothelial growth factor receptors (VEGFRs) with potent activity against KIT and PDGFA, has proven effective as a second-line therapy and is currently approved worldwide for metastatic GIST in patients with imatinib resistance or intolerance [7, 8]. Unlike imatinib mesylate, sunitinib has the ability to penetrate the BBB [8, 27, 28], making this the treatment of choice when GIST is not amenable to surgery or the patient has uncontrolled lesions.

Although radiotherapy is not a standard therapy for GIST, Cuaron et al. described the importance of this option for the management of locally advanced and metastatic GISTs [29]. In a series of 18 patients treated with radiotherapy, the estimated 6-month local progression-free and overall survival rates were 57.0% and 57.8%, respectively. For all patients, at least partial palliation was achieved in 17 patients (94.4%), and symptoms were completely alleviated in eight patients (44%). They concluded that radiotherapy can provide both objective responses and symptomatic relief without detracting dramatically from quality of life and should be considered in the multidisciplinary care of patients with locally advanced or metastatic GIST.

Despite the limitation of therapeutic strategies available, our findings remain hypothesis generating and offer important insights into a treatment modality that appears safe and effective for the management of patients with metastatic or locally advanced GIST.

## Figures and Tables

**Table 1:** List of published cases of metastatic gastrointestinal stromal tumors (GISTs) in the central nervous system (CNS).

Number	Age (years)	Sex	Primary site	Systemic metastasis	CNS site	Size of CNS tumor (cm)	Interval between diagnosis of primary and diagnosis of CNS metastasis	Treatment for CNS tumor	Mutational status	Survival prognosis after diagnosis of CNS metastasis	References
1	75	M	Mesentery	Liver	Both hemispheres	Infiltrative	14 months	Imatinib 800 mg/day	NA	CR (4 months)	Brooks et al. (2002) [12]

2	70	M	Stomach	Lung	Left occipital lobe	5.5	10 years	Total resection, radiation	NA	8 months	Inage et al. (2002) [17]

3	47	M	Jejunum	Liver	Left parasagittal	ND	25 months	Total resection, imatinib 800 mg/day	KIT (exon 9)	35 months	Hughes et al. (2004) [16]

4	60	M	Small intestine	Lumbosacral vertebrae (L5-S1)	Left cavernous sinus	ND	7 years	Radiation (54 Gy)	NA	8 months	Akiyama et al. (2004) [9]

5	76	M	Duodenum and jejunum	ND	Right parietal, right cerebellar hemisphere	2	4 months	Imatinib 400 mg/day, radiation (40 Gy)	No mutation in KIT (exon 11)	4 months	Kajikawa et al. (2005) [19]

6	68	F	Perisacral	ND	Right parietal lobe	3	2 years	Total resection, imatinib 800 mg/day	NA	CR	Kaku et al. (2006) [20]

7	42	M	Mesentery	ND	Right parietal lobe	3.5	Mesentery lesion later discovered	Total resection, radiation (60 Gy), imatinib 600 mg/day	NA	10 months	Puri et al. (2006) [21]

8	45	M	Small intestine	ND	Pontomedullary junction, cerebellum, leptomeningeal	2	5 years	Imatinib 800 mg/day	NA	2 months	Gerin et al. (2007) [15]

9	49	F	Mesentery	Liver	Left eye, brain	0.24	3 years	Imatinib 400 mg/day	NA	9 months	Gentile et al. (2008) [14]

10	54	F	Esophagus	Liver	Left frontal lobe	5	6 years	Total resection, imatinib 400 mg/day, SRT	KIT (exon 11)	CR (6 months)	Hamada et al. (2010) [10]

11	77	M	Jejunum	ND	Right cerebral peduncle, left occipital lobe	2.4 2.2	Jejunum lesion later discovered	Total resection, radiation (39 Gy), imatinib 400 mg/day	No mutation in KIT (exons 11, 13, 17, and 19), and PDGFR*α*	4 months	Naoe et al. (2011) [11]

12	26	M	Duodenum	Liver	Left frontotemporal	6.1 × 4.1	6 years	Total resection, radiation	NA	CR (4 months)	Wong and Chu (2011) [22]

13	15	M	Stomach	Liver	Right frontoparietal	4.2 × 3.3	12 years	Total resection, sorafenib 800 mg/day, sunitinib 37.5 mg/day	No mutation in KIT (exons 9, 11, 13, and 17), and PDGFR*α* (exons 12, 14, and 18)	CR (6 months)	Jagannathan et al. (2012) [18]

14	74	M	Jejunum	Liver	Right prefrontal gyrus	1.5 × 1.4	6 years	Sunitinib 50 mg/day, SRS	NA	CR (9 months)	Takeuchi et al. (2013) [8]

15	57	M	Stomach	ND	Left cerebellar, Left frontal	3	13 months	Total resection, SRS (18 Gy)	NA	CR (15 months)	Drazin et al. (2013) [13]

16	80	M	Small intestine	Cardiac apex, subclavial	Cerebellar vermis, Right frontal	4	Small intestine lesion later discovered	Total resection, RT (22 Gy)	NA	4 months	present case

CR, complete remission; F, female; M, male; NA, not analyzed; ND, not described; SRS, stereotactic radiosurgery; SRT, stereotactic radiotherapy.
